# Concise Review: Paracrine Role of Stem Cells in Pituitary Tumors: A Focus on Adamantinomatous Craniopharyngioma

**DOI:** 10.1002/stem.2267

**Published:** 2016-01-13

**Authors:** Juan Pedro Martinez‐Barbera, Cynthia L. Andoniadou

**Affiliations:** ^1^ Developmental Biology and Cancer Programme, Birth Defects Research Centre, Institute of Child HealthUniversity College LondonLondonUnited Kingdom; ^2^ Craniofacial Development and Stem Cell BiologyKing's College LondonGuy's CampusLondonUnited Kingdom

**Keywords:** Pituitary, Stem cells, Sox2, Tumors, WNT pathway

## Abstract

The existence of tissue‐specific progenitor/stem cells in the adult pituitary gland of the mouse has been demonstrated recently using genetic tracing experiments. These cells have the capacity to differentiate into all of the different cell lineages of the anterior pituitary and self‐propagate in vitro and can therefore contribute to normal homeostasis of the gland. In addition, they play a critical role in tumor formation, specifically in the etiology of human adamantinomatous craniopharyngioma, a clinically relevant tumor that is associated with mutations in *CTNNB1* (gene encoding β‐catenin). Mouse studies have shown that only pituitary embryonic precursors or adult stem cells are able to generate tumors when targeted with oncogenic β‐catenin, suggesting that the cell context is critical for mutant β‐catenin to exert its oncogenic effect. Surprisingly, the bulk of the tumor cells are not derived from the mutant progenitor/stem cells, suggesting that tumors are induced in a paracrine manner. Therefore, the cell sustaining the mutation in β‐catenin and the cell‐of‐origin of the tumors are different. In this review, we will discuss the in vitro and in vivo evidence demonstrating the presence of stem cells in the adult pituitary and analyze the evidence showing a potential role of these stem cells in pituitary tumors. Stem Cells *2016;34:268–276*

**Significance Statement**

In this concise review, we aim to discuss the accumulated evidence suggesting the presence of PSCs within the adult pituitary gland and the potential role of these cells in normal organ homeostasis. Additionally, we will elaborate on the data supporting the existence of CSCs in human and mouse tumours with particular emphasis to the role of pituitary PSCs in the aetiology of human craniopharyngioma.

## Introduction

In recent years, solid evidence has emerged for the existence of a tissue‐specific population of undifferentiated progenitors or stem cells within the hormone‐secreting anterior pituitary gland (pituitary stem cells, PSCs). These long‐lived, undifferentiated cells have the capacity to directly commit or generate daughter cells of three main progenitor lineages, characterized by respective expression of three transcription factors, PIT1 (POU1F1), TPIT (TBX19), and SF1 (NR5A1). Expression of these factors is a necessary step toward differentiation into a hormone‐secreting cell in the pituitary, where: PIT1‐positive cells give rise to GH‐expressing somatotrophs, PRL‐expressing lactotrophs, and TSH‐expressing thyrotrophs (PIT1‐cell lineage); TPIT‐positive cells give rise to ACTH‐expressing adrenocorticotrophs and melanotrophs; and finally, SF1‐positive cells give rise to LH‐ and FSH‐expressing gonadotrophs. The precise regulation of these populations ensures effective homeostasis and is dynamic throughout life, largely dependent on physiological demand. This can also be influenced by damage to pituitary tissue or to any of its target endocrine organs, and can be affected during disease states.

Similar to organ‐specific stem cells, analyses of many tumors and cancers have revealed the presence of multipotent cells, often thought to drive tumor formation. Conceptually, cancer stem cells (CSCs) are characterized by two features: (a) self‐renewal and (b) capability to generate the mass of the tumor/cancer cells in a cell‐autonomous manner (i.e., the tumor cells are descendants of the CSCs). This definition does not relate to their potential origin, whether these are normal tissue‐specific somatic stem cells, progenitors or differentiated cells. Experimentally, CSCs are determined by the identification of specific cells able to propagate a tumor/cancer and replicate its cellular composition in a relevant experimental setting, ideally in situ, either by lineage tracing or orthotopic transplantation. These have been isolated from leukemia and solid tumors 1, 2, 3, 4, 5. Generally, CSCs represent a small population of the tumor mass but are able to drive tumorigenesis when transplanted into a host, usually immunosuppressed mice (e.g., nonobese diabetic/severe combined immunodeficiency [NOD/SCID] mouse) 6. CSCs are endowed with molecular and cellular properties that make them particularly resistant to common anti‐cancer agents and radiotherapy 7, 8. They are often, although not necessarily, slow cycling or quiescent (nondividing) cells 9, and therefore antiproliferative agents or radiotherapy have little or no effect despite causing shrinking of the tumor mass 10. Similarly, CSCs express high levels of proteins that confer resistance to cytotoxic compounds such as anti‐cancer drugs (e.g., ABC transporters, and aldehyde dehydrogenase) 11. It is generally thought that effective anti‐cancer therapies should aim to eliminate the bulk of the tumor cells as well as the resident CSCs.

Many of the properties of CSCs are shared by normal tissue‐specific stem cells; slow cycling status, self‐renewal, and differentiation capacity and even resistance to cytotoxic drugs. For some tumors, it has been shown that normal stem cells are transformed into CSCs when targeted to express oncogenic proteins. For instance, stem cells of the intestinal crypt become CSCs when the WNT/β‐catenin pathway is over‐activated 12, 13. However, progenitor cells or even differentiated cells could give rise to CSCs upon oncogenic transformation 6, 14.

In this concise review, we aim to discuss the accumulated evidence suggesting the presence of PSCs within the adult pituitary gland and the potential role of these cells in normal organ homeostasis. Additionally, we will elaborate on the data supporting the existence of CSCs in human and mouse tumors with particular emphasis to the role of pituitary PSCs in the etiology of human craniopharyngioma. For more detailed discussion, we refer the reader to other recent reviews 15, 16, 17, 18, 19

## Cellular and Molecular Properties of PSCS

**Figure 1:**
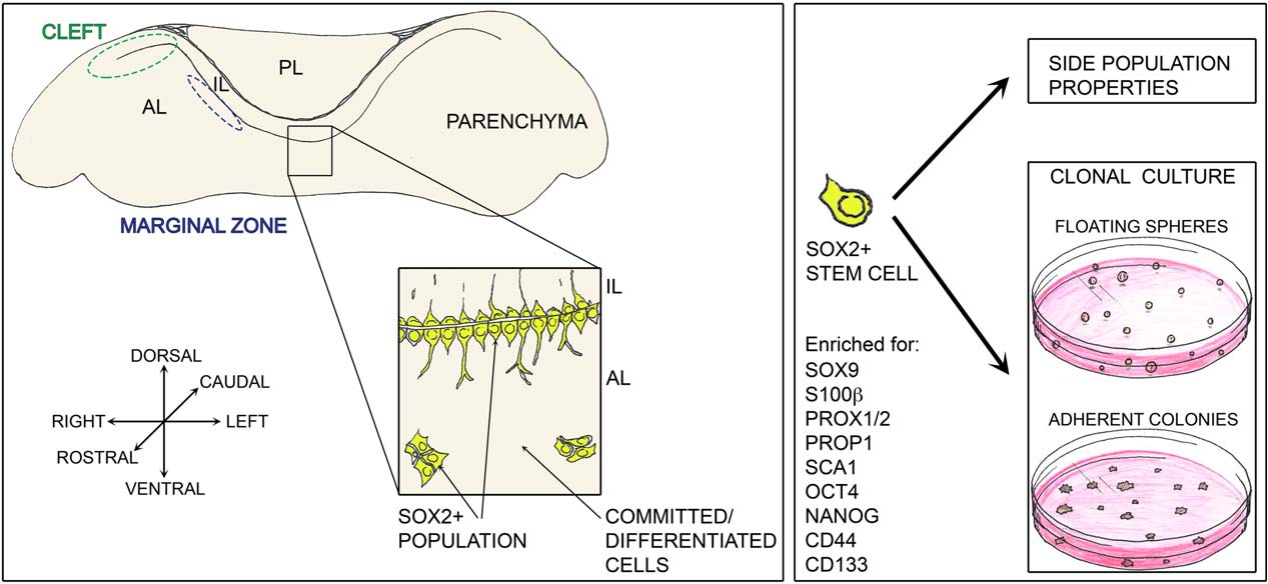
Stem cells in the anterior lobe of the mouse anterior pituitary. Pituitary stem cells (PSCs) reside within the SOX2 + population (yellow cells), which in the anterior lobe are located in the marginal zone epithelium at the interface with the intermediate lobe and enriched at the cleft, the lateral edges of the epithelium. SOX2 + cells are additionally found throughout the anterior lobe parenchyma. PSCs are enriched through efflux studies enabling isolation of a side population and are defined by their clonogenic capacity in vitro, cultured either as floating spheres or adherent colonies. A number of markers enriched in PSCs have been proposed; some of the commonly used are summarized here. Abbreviations: IL, intermediate lobe, AL, anterior lobe, PL, posterior lobe.

Two initial studies revealed postnatal mouse pituitary populations with in vitro self‐renewal capabilities; one population was shown capable of forming adherent colonies, and was characterized by the uptake of the fluorescent dipeptide β‐Ala‐Lys‐N ε AMCA (AMCA is 7‐amino‐4‐methylcourmarin‐3‐acetic acid) and included cells expressing S100 calcium‐binding protein B (S100β) 20, which have been described as folliculostellate cells of the anterior lobe 21. The other population was shown to form nonadherent spheres under clonal conditions in culture and characterized by marker expression relevant to stem cells of other tissues: expression of *Sca1, Oct4*, and *Nanog* 22. The same group later showed that cells within this population were enriched for SOX2, SOX9, CD44, and CD133 23. These cells had the capacity to efflux the vital dye Hoechst 33342, mainly through ABC transporter function, generating a “side population” during flow cytometry. This is typical of many cell types with stem/progenitor properties and the side population has been described for pituitaries of other vertebrates 22, 24, 25. Since these early characterizations, many additional markers of cells with in vitro clonogenic capacity (another characteristic of stem cells) have been proposed, such as Nestin, PROP1, SOX9, GFRα2, and PRX1/2 26, 27, 28, 29, 30. The transcription factor SOX2 is highly associated with pluripotency in the early embryo, in embryonic stem cells (ESCs) and induced pluripotent stem cells (iPSCs), as well as the stem cell state in multiple embryonic and adult tissues. In the pituitary, it was shown to define a population, which exclusively contains cells capable of both adherent and nonadherent clonogenic colony formation (Fig. 1) 31, 32. In vivo in the postnatal rodent gland, SOX2‐positive cells show a high degree of overlap with other proposed stem cell markers such as PROP1 27, 33, PRX1/2 30, and SOX9 28.

Although SOX2‐positive cells do not express any hormone and SOX2 expression does not overlap with expression of the committed progenitor markers, the population has a heterogeneous nature in terms of properties, where only a low proportion (2.5%–5%) are shown to be capable of in vitro self‐renewal. This suggests that perhaps not all SOX2‐positive cells in the pituitary have stem cell capacity, or that our current assay methodologies select a subpopulation, perhaps at a particular primed state or phase of the cell cycle, and maybe the remainder of the SOX2‐positive cells would flourish under alternative conditions. Both cases highlight the need for better marker characterization, whose overlapping combination would possibly define the true pituitary stem cell, poised for activation. Purifying pituitary cells based on S100β expression using S100β‐GFP transgenic animals enriches in vitro colony formation, a property lying exclusively within the SOX2‐positive component 34, hence suggesting that the overlapping population is likely to be enriched in stem cells and the strong association of S100β with multiple proposed pituitary stem cell populations makes this protein a good candidate marker. Indeed, there is significant overlap between SOX2 and S100β positivity 31, 34 (Fig. 1). On its own, S100β marks multiple populations, displaying expression in the SOX2‐positive marginal zone region, in the morphologically distinct folliculostellate cells and reported to be expressed in pituicytes. In rat, at least two distinct S100β populations have been isolated, one with processes typical of folliculostellate cells, which is positive for GFAP and vimentin and a second, morphologically rounded cell type lacking processes, which express dendritic markers 35. Multiple functions have been put forward for S100β‐positive folliculostellate cells reviewed comprehensively elsewhere 36, including providing structural support and enabling communication within the pituitary facilitating pulsatile hormone secretion 37, 38. A further attribute of these cells is the production of paracrine signals that contribute to the regulation of surrounding endocrine cells. For example, synthesis and secretion of FSH in gonadotrophs is mediated by activin, and regulated by the activin‐binding protein follistatin, both of which are produced by folliculostellate cells 39. Numerous other paracrine factors secreted by these cells have been identified, including interleukin‐6 (IL‐6), leukemia inhibitory factor (LIF), vascular endothelial growth factor (VEGF), and basic fibroblastic growth factor (bFGF), all of which contribute to pituitary function 40, 41, 42.

The location of cells expressing SOX2/S100β has also been proposed to enable stem cell function. The marginal zone, lining the remnants of Rathke's pouch lumen, retains an epithelium of SOX2^+^ cells on either side. Additionally, SOX2^+^ cells are found in the parenchyma of the anterior pituitary, often in groups distributed among hormone‐secreting cells (Fig. 1). The in vitro clonogenic potential of SOX2 cells does not differ between the two locations 35 and it has been postulated that these cells form a network spanning the whole pituitary, making connections through their projections 43. Recent studies from rat have revealed that SOX2^+^/S100β^+^ double‐expressing cells include a further subset that express the gene *Cxadr* in vivo, which codes for coxsackievirus and adenovirus receptor (CAR), that facilitates formation of homophilic tight junctions 44. Furthermore, expression of E‐cadherin and the juxtacrine factor ephrin‐B2 reportedly define SOX2^+^/S100β^+^/CAR^+^ cells, both in the marginal epithelium and throughout the parenchyma 44, 45. Analysis of the side population by the group of Vankelecom had also reported enrichment in ephrin‐B expression in this stem cell‐rich compartment 46.

## Contribution of Stem Cells in the Long‐Term Maintenance of the Anterior Pituitary

Despite a plethora of identified markers, until recently, there was no evidence to support that pituitary stem cells function as such in vivo. This changed with the generation of two similar genetic tools, inducible mouse strains expressing CreERT2 under the regulation of the SOX2 promoter generated by the Hochedlinger and Martinez‐Barbera/Pevny labs 34, 47. In these, Cre is expressed in cells expressing *Sox2*, but will not be active until the administration of tamoxifen, allowing temporal control of recombinase action. Although the expression construct of these two strains is not identical, the expression of CreERT2 faithfully reproduces that of *Sox2* in both. We used one of these mouse strains to lineage trace cells expressing *Sox2* beginning at different stages, both during gestation and postnatally 34. Similarly, the Lovell‐Badge group used the strain generated by the Hochedlinger laboratory to trace *Sox2*‐expressing cells from embryonic stages 28. In all cases, *Sox2*‐expressing cells gave rise to all committed progenitor cell types (PIT1, TPIT, SF1), and thus all hormone‐secreting cells types of the anterior lobe (GH, PRL, TSH, ACTH, LH/FSH). One limitation of this assay is that it does not distinguish between a multipotent population of *Sox2*‐expressing cells and the possibility that several oligopotent or unipotent populations of *Sox2*‐expressing cells exist, collectively giving rise to all differentiated lineages. To demonstrate that the population of SOX2^+^ cells is not depleted, as would be expected by transit‐amplifying progenitor cells, we activated CreERT2 in SOX2^+^ cells by postnatal tamoxifen administration, enabling expression of *R26R‐EYFP* and cells were traced for 6 months. At the end of this period, descendants of SOX2^+^ cells were flow sorted for EYFP expression and cultured to assess clonogenic potential, a property contained only among SOX2^+^ cells. Most of the cells with clonogenic potential were residing in the EYFP positive fraction, suggesting that SOX2 cells are either long‐lived hence persisting after their initial labeling, or maintained as a self‐renewing pool of stem cells derived from the originally labeled SOX2^+^ cells. If SOX2^+^ cells were a population of transit amplifying cells with short‐term uncommitted proliferative potential, we would expect that this population would become depleted and lose properties associated with the stem cell state, such as clonogenic capacity, following their commitment/differentiation. Complementing this, following postnatal tamoxifen inductions we found a significant population of uncommitted SOX2^+^ and SOX9^+^ cells up to a year following tamoxifen administration. The above experiments demonstrate the presence of a long‐lived population that retains pituitary stem cell properties throughout normal life.

## Stem Cells from Pituitary Tumors

Several groups have reported the presence of putative CSCs in pituitary adenomas isolated from mice and humans 18, 48, 49, 50, 51, 52, 53, 54, 55. The criteria for a cell to be termed a CSC are based on some or all of the following properties: (a) self‐propagation in vitro (clonogenic potential); (b) multipotent differentiation capacity; (c) expression of “stemness” markers; (d) chemoresistance; (e) tumorigenic potential in transplantation experiments.

**Figure 2:**
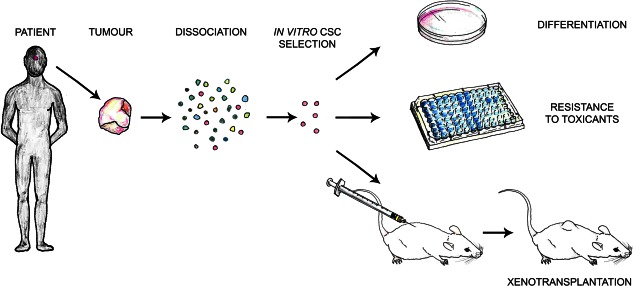
Methods of cancer stem cell characterization in human pituitary adenoma. Fresh tumor tissue can be dissociated and cancer stem cells (CSC) selected in vitro based on culture properties and marker expression. Assays to determine CSC potential are in vitro differentiation to determine their multipotent nature for appropriate tissue lineages, assays to determine possible resistance to cytotoxic compounds (characteristic of CSCs), and xenotransplantation into animal models where CSCs are expected to reinitiate tumor growth. Abbreviation: CSC, cancer stem cell.

A summary of the experimental approach used is as follows (Fig. 2): tumors are dissociated into single cell suspensions and cultured in vitro in stem cell‐promoting media, which contains fibroblast growth factor (FGF) and epidermal growth factor (EGF) but no serum. After a few days, floating spheres emerge, which can be passaged several times and forced to differentiate into hormone‐producing cells when cultured in media supplemented with serum and/or hypothalamic stimulating factors controlling anterior pituitary function, and in the absence of growth factors. In some studies, the side population assay has been used to purify tumor cells able to efflux Hoechst dye, enriching for potential CSCs 17, 23, 56. These resulting tumor‐derived spheres express markers associated with stemness (e.g., Nestin, SOX2, SCA1, and CD133) and do not express differentiation markers (e.g., growth hormone). In one study, it has been shown that the undifferentiated cells contained in the spheres are more resistant to chemotherapeutics than differentiated cells 49. These in vitro‐grown spheres contain tumor‐propagating cells capable to generate tumors when transplanted into the brain 49, 50 or under the skin 48 of immunosuppressed mice. In one study, grafted tumors have been proven to be serially transplantable 49.

Overall, these reports have provided evidence supporting the existence of CSCs with tumor‐propagating capacity. However, several questions remain unanswered, among them: (a) Do CSCs arise from PSCs? PSCs and CSCs share similar properties, for instance in vitro self‐propagation and differentiation potential, and it is likely that PSCs are also chemoresistant, since a side population that is enriched for PSCs can be purified from normal mouse pituitaries using the vital dye Hoechst efflux assay 23. (b) Can CSCs generate pituitary tumors when transplanted orthotopically in the pituitary of host mice? Orthotopic transplantations have not been performed due to technical difficulties. These experiments will reveal whether CSCs can be tumorigenic in a normal pituitary gland environment. Related to this latter question, (c) can normal PSCs generate tumors when transplanted heterotopically in the brain? Human PSCs may be difficult to obtain, but mouse PSCs are readily available.

In our opinion, questions (b) and (c) are very relevant because they will assess the effects of the culture conditions and cellular microenvironment on the tumorigenic capacity of normal PSCs and of CSCs. Although generally speaking normal stem cells do not form tumors, the potential of tumor development is considered as one of the risks of the use of stem cells in regenerative medicine 57, 58. Normal hematopoietic stem cells do not form tumors when injected into recipient subjects, however, the evidence for other somatic stem cells is not as solid and there are cases where “normal donor‐derived somatic stem cells” have been found to be the origin of cancers in transplanted human and mouse hosts 59, 60, 61. Normal untransformed PSCs could acquire genetic or epigenetic alterations during their culture period, which may predispose them to form tumors, as described for other stem cells 62. The local environment in which the stem cell resides may also influence its tumorigenic potential. The concept of how the cellular environment can affect the potential tumorigenic effects of cells is beautifully exemplified by the capacity of normal mouse and human embryonic stem cells to generate either teratomas, when heterotopically transplanted into adult mouse tissues, or normal chimeric organs, when transplanted into developing embryos 63, 64, 65.

Similarly, the expansion of clonogenic pituitary CSCs before transplantation that has been carried out in all published reports could select for specific cell populations present in the tumor or even induce genetic or epigenetic changes that could account for the tumorigenic effect described. Fresh cell suspensions from pituitary tumors that have not been cultured in vitro should also be tested in transplantation experiments. In this regard, a recent report has shown how critical the influence of the in vitro manipulations may be on the final outcome of the transplantations. Human glioma tumor cells cultured in stem cell‐promoting medium generate tumors of human origin in orthotopic xenotransplantations, whilst fresh cell suspensions induce tumors of mouse origin, suggesting a paracrine influence on the host tissue 66. Perhaps, future mouse experiments should also address the tumorigenic potential of “normal” PSCs when transplanted heterotopically.

## Paracrine Contribution of Stem Cells to Tumorigenesis

Recent studies of a type of pituitary tumor called adamantinomatous craniopharyngioma (ACP) have revealed a critical role for mutated PSCs in promoting tumorigenesis through paracrine activities.

ACPs are aggressive tumors of the sellar region due to their tendency to infiltrate the brain, optic pathways and nearby vascular structures 67. Although it can affect adults, ACP is mostly a pediatric tumor and represents the most common pituitary tumor in children 68. Mutations in *CTNNB1*, the gene encoding β‐catenin, have been identified in the majority of ACP samples 69. These mutations are predicted to interfere with β‐catenin degradation resulting in the accumulation of the protein and the over‐activation of the WNT/β‐catenin pathway 70. Indeed, immunostaining analysis has revealed the presence of a small population of cells accumulating nucleocytoplasmic β‐catenin, the hallmark of pathway activation, either dispersed throughout the tumor or grouped forming cell clusters 71. These clusters are a typical histological finding of human ACP that is not observed in any other pituitary tumor 72. Studies in mouse models have provided important insights into the etiology and pathogenesis of human ACP.

It has been shown that the expression of a degradation‐resistant form of β‐catenin in Rathke's pouch (RP) precursors in the mouse (*Hesx1^Cre/+^; Ctnnb1^lox(ex3)/+^* mouse model) is sufficient to form tumors that are very similar to human ACP 73. RP is the primordium of the anterior pituitary and contain undifferentiated precursors capable of self renew transiently and generate all of the hormone‐producing cells of the anterior pituitary. As the human ACP, mouse tumors contain nucleocytoplasmic‐accumulating cell clusters that activate the WNT/β‐catenin pathway. Moreover, gene profiling analysis has shown cell clusters are likely to represent similar structures in human and mouse ACP 32.

Human ACPs have not been analyzed for the presence of CSCs as previously described for human adenoma. Reports have suggested the presence of stem cell marker expression in human ACPs 74, 75, but so far no functional characterization has been performed. If CSCs exist in these benign tumors, the mutational landscape seems to be very restricted as no recurrent mutations have been found in addition to those in β‐catenin. There have been rare cases of malignant ACP, possibly linked to the use of radiotherapy 76, 77, 78. In these malignant neoplasms p53 is over‐expressed 79, perhaps indicating that other mutations may have been induced as the result on the irradiation generating more aggressive CSCs.

Mouse ACPs, however, have been shown to contain cells capable of self‐propagation and differentiation properties in vitro 73. Relative to normal pituitary glands, mouse ACPs contain up to threefold higher numbers of clonogenic cells when cultured in stem cell‐promoting medium, suggesting an enlargement of the stem cell compartment in these tumors. These cells express stemness markers such as *Sox2* and *Nestin*, and show very low levels of expression of differentiation markers. Moreover, time‐lapse microscopy has revealed that the proliferation rate of clonogenic cells in the tumors is elevated 1.7‐fold compared with normal PSCs isolated from control pituitaries. Whether these clonogenic cells can generate tumors when transplanted remain to be tested.

**Figure 3:**
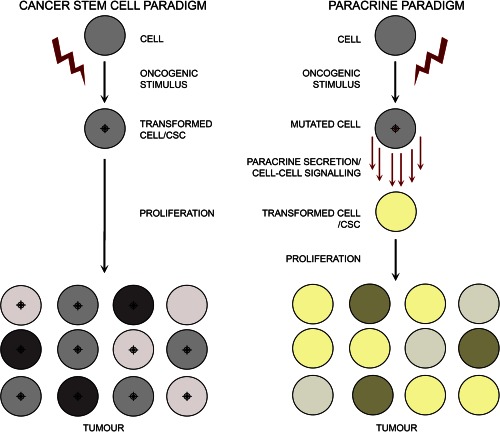
Schematic comparing two models of tumor formation: the cancer stem cell and paracrine paradigms. In the cancer stem cell paradigm (left), a cell, which may be a stem cell, committed or differentiated cell becomes transformed into a cancer stem cell (CSC) upon exposure to an oncogenic stimulus. This transformed CSC proliferates to give rise to direct derivatives that compose the tumor mass. In the paracrine paradigm, upon receipt of the oncogenic stimulus the cell becomes secretory. The paracrine signals emanating from this mutated cell can be perceived by a competent surrounding cell that will become transformed to act like a CSC and become the cell‐of‐origin of the tumor. Abbreviation: CSC, cancer stem cell.

Further studies in mice, aiming to understand the role of PSCs in generation of ACP tumors have been performed. Expression of oncogenic β‐catenin specifically in SOX2^+^ stem cells of the adult pituitary (*Sox2^CreERT2/+^; Ctnnb1^lox(ex3)/+^* mouse model) also results in tumors that are similar to human ACP 34. These mouse tumors contain cell clusters showing nucleocytoplasmic β‐catenin and are undifferentiated (i.e., do not express any hormone), as the human tumors. This mouse model has been used to trace the descendant cells of the mutated SOX2^+^ PSCs, a technique referred to as “genetic tracing” 80. SOX2^+^ cell are targeted to express simultaneously oncogenic β‐catenin and yellow fluorescent protein (YFP), the former is the oncogenic stimulus and the latter allows identification of all the daughter cells derived from the mutated SOX2^+^ PSCs. These experiments have revealed that the typical cell clusters derive from SOX2^+^ cells, but intriguingly, the bulk of the tumor mass does not. In other words, mutated SOX2^+^ cells are not transformed into CSCs by oncogenic β‐catenin; mutated SOX2^+^ PSCs generate cell clusters that have the capacity to induce tumors in a paracrine fashion so that the tumor cells are not derived from SOX2^+^ cells 34 (Fig. 3, paracrine paradigm). Although not proven, it is possible that the paracrine activities of the clusters induce CSCs, which become the cell‐of‐origin of the tumors. In agreement with this notion, cluster cells express a vast array of growth factors, chemokines and cytokines, and are acting as signaling centers, possibly changing the tumor microenvironment and facilitating tumorigenesis 32, 34. Of relevance, expression of several cytokines and growth factors have been shown to play a role in normal pituitary physiology as well as in pituitary adenoma, a more prevalent pituitary tumor in humans 40, 81, 82.

## Broader Implications of the Paracrine Model of Tumorigenesis in the Cancer Field

CSCs carry mutations in oncogenes and tumor supressors, which are mostly responsible for their tumorigenic behavior. The identification of these mutations in human neoplasms is of much clinical relevance, as this is the first step toward the development of specific therapies to kill these cells that drive neoplasia. The better understanding of the early steps that lead to the generation of CSCs is also relevant, as this may lead to development of early diagnostic tools that would allow the treatment of human neoplasms at incipient stages, possibly where they are more vulnerable to anti‐cancer therapies. The pertinent question is what drives the oncogenic transformation and generation of CSCs?

An excess of growth factors/mitogenic signals may lead to cell transformation. For instance, by over‐activating the same pathways affected by oncogenic mutations (e.g., β‐catenin, BRAF, KRAS, PATCH1 among others) resulting in increased cell proliferation, DNA replicative stress, DNA damage, and eventually genetic mutations conferring tumorigenic capacity in transformed cells. Compatible with this notion, the synthesis and secretion of human TGF‐alpha by rat fibroblasts results in the loss of anchorage‐dependent growth in vitro and tumor formation in nude mice 83. Similarly, over‐expression of platelet‐derived growth factor (PDGF) in neural precursors is sufficient to induce brain tumors resembling human glioma 84, 85; basic fibroblast growth factor (bFGF) 86 can also transform normal cells resulting in tumors. This may apply to many more growth factors including EGF, insulin, and hepatocyte growth factor (HGF) among others 87.

Genetic evidence in mouse has also shown that tumors/cancers can develop in a noncell autonomous manner, whereby the bulk of the tumor mass consists of cells that do not carry the oncogenic mutations. For instance, conditional deletion of *Notch1* in mouse skin epidermis 88 or hair follicles stem cells 89 leads to overactivation of the WNT/β‐catenin pathway and induction of tumors in a noncell autonomous manner (i.e., the tumor cells are wild type for *Notch1*). Additionally, the expression of a constitutively active form of MEK1, activating the Erk mitogen‐activated protein kinase (MAPK) pathway, in mouse epidermis results in skin polyps which are mostly formed by wild‐type cells for MEK1 90. These examples, however, do not demonstrate cellular transformation or tumor‐propagating potential of the cells that do not carry the oncogenic hit.

Two recent manuscripts have shown that cell transformation can occur in a paracrine manner in vivo. In a hepatocellular carcinoma mouse model, it was demonstrated that deletion of the tumor suppressor gene p53 specifically in hepatic stellate cells induces epithelial tumors that are mostly wild type for p53 91. More strikingly, in a mouse model for leukemia the expression of a degradation resistant form of β‐catenin (i.e., the same used to generate mouse ACP) in osteoblast precursors is sufficient to generate oncogenic hematopoietic stem cells (HSCs) capable of giving rise to acute myeloid leukemia (AML). Moreover, transplantation of wild‐type bone‐marrow cells to lethality irradiated mice expressing mutant β‐catenin in osteoblasts also results in AML, suggesting a critical role of the niche environment in cell transformation. Furthermore, transplantation of long‐term repopulating HSC progenitors, but not other hematopoietic populations, can propagate AML when transplanted into wild‐type hosts, indicating that they have become CSCs and do not require the paracrine activities of the osteoblasts 92.

We think that the studies cited above provide support to the potential tumor‐inducing properties of the β‐catenin‐accumulating cluster cells in mouse and human ACP. These cluster cells express members of the TGF, FGF, and PDGF families of growth factors among many others. In addition, the extracellular matrix (ECM) is also altered around the clusters 34. Therefore, cluster cells may be signaling to surrounding cells either through cell–cell interactions or secreted factors, with overstimulation of signaling pathways leading to changes in receptive cells. Circumstantially, actively proliferating cells stained by Ki67 immunostaining can be detected in close proximity to the cell clusters 73. Further research will reveal the mechanisms whereby cell clusters may induce paracrine cell transformation and promote tumor growth.

## Conclusions

Murine and human studies have provided support to the idea that CSCs play a role in pituitary tumorigenesis. Moreover, analyses of a mouse model for human craniopharyngioma have revealed that paracrine signaling may be critical in tumour initiation. It may be argued that paracrinicity is only of relevance to the understanding of benign tumours. However, once tumours progress into malignancy, cancer cells may be self‐sufficient and able to generate the trophic factors needed for survival and growth, independent of the initial signals that cause cell transformation. Even if this latter scenario is real, elucidating these initial stages of tumorigenesis will provide with a better grasp of the pathways that initiate these malignancies. Such knowledge could translate to the discovery of early diagnostic biomarkers, which could detect the cancer at incipient stages of development, when tumour cells are more susceptible to anti‐cancer treatments. Efforts to better characterise and understand the potential of normal stem cells of the pituitary gland can only enhance our ability to comprehend their possible role in pituitary tumour formation and propagation, which can lead to more effective prognosis and treatments.

## Author Contributions

J.P.M.B. and C.L.A. wrote the manuscript and approved the final version for publication.

## Disclosure of Potential Conflicts of Interest

The authors indicate no potential conflicts of interest.
